# Support vector machine-based classification of schizophrenia patients and healthy controls using structural magnetic resonance imaging from two independent sites

**DOI:** 10.1371/journal.pone.0239615

**Published:** 2020-11-24

**Authors:** Maeri Yamamoto, Epifanio Bagarinao, Itaru Kushima, Tsutomu Takahashi, Daiki Sasabayashi, Toshiya Inada, Michio Suzuki, Tetsuya Iidaka, Norio Ozaki

**Affiliations:** 1 Department of Psychiatry, Nagoya University, Graduate School of Medicine, Nagoya, Aichi, Japan; 2 Brain & Mind Research Center, Nagoya University, Nagoya, Aichi, Japan; 3 Medical Genomics Center, Nagoya University Hospital, Nagoya, Aichi, Japan; 4 Department of Neuropsychiatry, University of Toyama Graduate School of Medicine and Pharmaceutical Sciences, Toyama, Toyama, Japan; Hamamatsu University School of Medicine, JAPAN

## Abstract

Structural brain alterations have been repeatedly reported in schizophrenia; however, the pathophysiology of its alterations remains unclear. Multivariate pattern recognition analysis such as support vector machines can classify patients and healthy controls by detecting subtle and spatially distributed patterns of structural alterations. We aimed to use a support vector machine to distinguish patients with schizophrenia from control participants on the basis of structural magnetic resonance imaging data and delineate the patterns of structural alterations that significantly contributed to the classification performance. We used independent datasets from different sites with different magnetic resonance imaging scanners, protocols and clinical characteristics of the patient group to achieve a more accurate estimate of the classification performance of support vector machines. We developed a support vector machine classifier using the dataset from one site (101 participants) and evaluated the performance of the trained support vector machine using a dataset from the other site (97 participants) and vice versa. We assessed the performance of the trained support vector machines in each support vector machine classifier. Both support vector machine classifiers attained a classification accuracy of >70% with two independent datasets indicating a consistently high performance of support vector machines even when used to classify data from different sites, scanners and different acquisition protocols. The regions contributing to the classification accuracy included the bilateral medial frontal cortex, superior temporal cortex, insula, occipital cortex, cerebellum, and thalamus, which have been reported to be related to the pathogenesis of schizophrenia. These results indicated that the support vector machine could detect subtle structural brain alterations and might aid our understanding of the pathophysiology of these changes in schizophrenia, which could be one of the diagnostic findings of schizophrenia.

## Introduction

Schizophrenia is a detrimental psychiatric disorder characterized by positive symptoms (delusions and hallucinations), negative symptoms (impaired motivation, reduction in spontaneous speech, and social withdrawal), and cognitive impairment (working memory deficit, attentional impairment) [1]. Structural brain alterations in schizophrenia have been repeatedly reported in the literature; however, the pathophysiology of such alterations remains unclear [1]. Voxel-based morphometry (VBM), a univariate analysis based on a voxel- or cluster-level comparison, has detected group level differences in gray matter densities between patients with schizophrenia and controls [2]. Meta-analyses based on VBM have also identified gray matter deficits in patients with schizophrenia, particularly in the frontal and temporal lobes, cingulate and insular cortices, and thalamus [3–5]. Gray matter deficits in multiple distributed brain regions have been implicated in schizophrenia by VBM studies, but the VBM method does not consider the interconnected nature of the brain regions [6].

To overcome these methodological disadvantages, an increasing number of studies have applied multivariate pattern recognition analysis (MVPA) to extract brain alterations in patients with schizophrenia [7]. Unlike univariate analysis, which considers each voxel independently, MVPA can detect subtle and spatially distributed patterns of structural alterations that are not detected by univariate analysis [8]. Furthermore, MVPA can be used to individually classify patients and healthy controls based on the identified pattern of brain alterations. Therefore, MVPA may be a potential tool to detect the pathophysiology of brain structural alterations in schizophrenia.

Support vector machine (SVM) is one of the popular methods of MVPA in the neuroimaging of psychiatric disorders [9]. The use of an SVM typically involves a training phase, in which datasets with known associations between group features and group membership are used to train a classifier to discriminate the different groups. Subsequently, the trained classifier is used to classify a new dataset in a testing phase for validation [9]. Validation using independent datasets is the appropriate method; however, this method requires a larger number of samples, which may not be readily available. If the sample size is limited, alternative methods such as a cross-validation approach can be performed to evaluate the SVM’s classification performance [10–12]. Although cross-validation can provide an unbiased estimate of the classifier’s performance, there is still a danger of overestimating the classification performance because the test dataset includes the training set [2]. In schizophrenia research, most previous studies have adopted the cross-validation method for validation [13], which limits the generalizability of the overall findings.

Another important question that needs to be fully addressed for clinical applicability of MVPA-based approaches is whether classifiers trained using data from one site can be effectively applied to data obtained from another site. The difficulties in multisite analysis is that the different MRI scanners and protocols can have an effect on the performance of the classifications [14, 15]. Until recently, most classification studies using two independent samples for training and testing obtained data from a single study site [16–18]. The performance of data from multi-site classification still remains unexplored. Rozycki et al. [19] addressed this issue and reported that consistent classification accuracy could be achieved even if the training and testing data were acquired from different sites.

In this study, we applied an SVM to classify patients with schizophrenia and control participants on the basis of structural MRI data. Unlike previous studies and following Rozycki et al. [19], to evaluate SVM classification performance, we used independent datasets obtained from different sites using different MRI scanners and protocols and enrolled patients with different clinical characteristics. This enabled us to examine the robustness and general applicability of the trained SVMs to classify patients from healthy controls across different sites and patient cohorts. Additionally, we investigated the patterns of structural alterations that significantly contributed to the classification performance of SVMs and examined how these changes were correlated to patients’ clinical characteristics.

## Methods

### Participants

The present study included two datasets, one from Nagoya University and the other from Toyama University in Japan. Table 1 presents the demographic and clinical data of the participants. The Nagoya University dataset consisted of 50 patients with schizophrenia and 51 healthy controls. The Toyama University dataset consisted of 49 patients and 48 healthy controls. The patients were diagnosed based on the Diagnostic and Statistical Manual of Mental Disorders, Fourth Edition diagnostic criteria [20] using the Structured interview. Current clinical symptom severity was assessed using the Positive and Negative Syndrome Scale (PANSS) [21]. The dose of antipsychotic medication received at the time of scanning was evaluated by the chlorpromazine (CPZ) equivalent [22]. The healthy controls had no history of psychiatric or neurological disorders (based on the Structured Clinical Interview for Diagnosis, non-patient version) [23] and did not use any psychoactive medications. Intelligence quotient scores were estimated using the Japanese version of the National Adult Reading Test (JART) [24]. Handedness was assessed using the Edinburgh Handedness Inventory [25] for participants from Nagoya University and the Rating Scale of Handedness for Biological Psychiatry Research among Japanese People [26] for participants from Toyama University. All procedures in this study were carried out in accordance with the Declaration of Helsinki; the participants provided written informed consent to participate; and the Nagoya University Graduate School of Medicine, Nagoya University Hospital Ethics Review Committee and the Committee on Medical Ethics of Toyama University approved this study.

**Table 1:** Demographic and clinical information.

	Nagoya University		Toyama University	
	SCZ (*n* = 50)	CON (*n* = 51)	SCZ (*n* = 49)	CON (*n* = 48)
Age at scan (years)	38.8 (± 6.9)	36.5 (±7.1)	28.1 (± 5.0)	26.9 (±3.3)
Sex (male/female)	26/24	29/22	23/26	23/25
Handedness (right/both/left)	49/0/1	51/0/0	32/2/9	36/2/9
Education	13.4 (2.7)	16.5 (1.5)	13.7 (2.1) ^†^	17.3 (1.4)
Estimated IQ (JART)	98.9 (10.2)	108.0 (6.4)	101.6 (10.2)^‡^	111.0 (5.9)
Onset age (years)	24.1 (6.4)		22.5 (5.1)	
Duration of illness (years)	14.7 (8.2)		5.4 (4.8)	
Dose of antipsychotics (CPZ equivalent) (mg)	584.4 (406.6)		441.9 (416.7) ^§^	
PANSS Total	67.3 (23.5)		64.6 (21.3) ^¶^	
PANSS Positive	16.0 (6.3)		13.4 (5.8)	
PANSS Negative	16.9 (6.8)		18.2 (7.9)	

Data are presented as the mean (standard deviation). Abbreviations: Schizophrenia (SCZ), healthy control (CON), intelligence quotient (IQ), Japanese version of the National Adult Reading Test (JART), chlorpromazine (CPZ), Positive and Negative Syndrome Scale (PANSS)

^†^ Information is missing for one patient.

^‡^ Information is missing for five patients.

^§^ Seven patients did not take antipsychotics.

^¶^ Information is missing for two patients.

### MRI acquisition

Participants from Nagoya University were scanned using a 3-T MRI scanner (Siemens, Verio, Erlangen, Germany) at the Brain & Mind Research Center, Nagoya University with the following parameters: repetition time (TR) = 1900 ms, echo time (TE) = 2.48 ms, inversion time (TI) = 900 ms, flip angle = 9°, field of view (FoV) = 256 mm, resolution = 256, number of slices = 192, slice thickness = 1.3 mm, and voxel size = 1 x 1 x 1.3 mm. The participants from Toyama University were scanned using a 3-T MRI scanner (Siemens Magnetom Verio, Erlangen, Germany) at Toyama University with the following parameters: TR = 2300 ms, TE = 2.9 ms, TI = 900 ms, flip angle = 9°, FoV = 256 mm, resolution = 256, number of slices = 178, slice thickness = 1.2 mm, and voxel size = 1 x 1 x 1.2 mm.

### MRI preprocessing

MRI data were processed using the VBM 8 toolbox provided by Christian Gaser (http://dbm.neuro.uni-jena.de/vbm.html) in the Statistical Parametric Mapping 8 program (http://www.fil.ion.ucl.ac.uk/spm/software/spm8/). The T1 images were normalized and segmented into gray matter, white matter, and cerebrospinal fluid using VBM 8’s unified segmentation process. The Diffeomorphic Anatomical Registration using Exponentiated Lie Algebra [27] was used to create a group template for spatial normalization of the segmented images of each participant. The modulation step was performed using exclusively non-linear regression. The normalized and modulated gray matter images were then spatially smoothed using an 8 mm full-width at half-maximum Gaussian filter. Age and sex were also regressed from the preprocessed gray matter images independently for each site using the Matlab’s *regress* function to account for any differences due to these covariates. These preprocessed images were then used in the succeeding SVM analysis.

From the preprocessed gray matter images, we also generated a mask by computing the mean gray matter image using all (both sites) participants’ data and applying a gray matter density threshold of 0.2. Voxels with values above the threshold were included in the mask. The resulting mask was then applied to all images in order to limit the number of voxels used for the classification.

### SVM classification

To classify patients from controls, we used a linear SVM. For the SVM analysis, we used in-house Matlab scripts and the Matlab version of LIBSVM, a library for support vector machines [28]. We used one dataset to train an SVM classifier and tested the performance of the trained classifier using the other dataset. The SVM was trained to classify gray matter images as either belonging to the patient group (assigned a class label of -1) or to the control group (assigned a class label of +1). The regularization parameter for the linear SVM was estimated using a ten-fold cross-validation approach. The trained SVM’s classification performance was assessed using the following measures: accuracy, sensitivity, specificity, positive predictive value, and negative predictive value.

After training, the SVM assigns weight values for each voxel, which can then be used to assess each voxel’s contribution to the SVM’s classification accuracy. To identify the significance of the weight value of each voxel, a permutation test [29] with 5000 iterations was used. This was achieved by randomly permuting the class labels, then training an SVM using the permuted labels to yield the probability distribution of the SVM weights under the null hypothesis of no association between class labels and the global structure of the training data. The p-value of each weight was then estimated as the proportion of weight values in the null distribution greater than or equal to the value obtained using the original (non-permuted) labels. A significance map was constructed by estimating the p-values of all voxels within the mask used in the analysis.

We trained SVM classifiers using the dataset from one site and evaluated the performance of the trained SVMs using the dataset from the other site, and vice versa. Several classification measures were assessed and the brain regions that significantly contributed to the classification of the two groups were identified using the constructed significance map.

### Correlation between gray matter densities in regions contributing to the classification performance of the trained SVMs and clinical features

We also assessed the association between the gray matter densities in brain regions that significantly contributed to the classification accuracy of the trained SVMs and patients’ clinical features such as onset age, duration of illness, CPZ equivalent, and JART in both models. For this, we extracted regions-of-interest (ROIs) from the significance map of each model by setting a threshold using a false discovery rate (FDR) of q < 0.05 and cluster size of more than 100 voxels. For each ROI, we then extracted the gray matter values from the preprocessed images, computed the mean within the ROI, and assigned the resulting value to the ROI. We assessed the relationship between the mean gray matter densities in the obtained ROIs and the clinical data of patients (JART, PANSS, onset age, duration of illness, and CPZ equivalent). This analysis was performed independently for each site since the SVM models are site-specific.

## Results

### Participant characteristics

Five patients were missing JART, one was missing education duration, and two were missing PANSS information in the patient group in the Toyama University dataset. Patients and controls did not differ significantly in terms of age or sex within each university [*t* (98) = -1.35, *p* = 0.18 and *χ*^*2*^ = 0.04, *p* = 0.84 in Nagoya University; and *t* (95) = -1.39, *p* = 0.17 and *χ*^*2*^ = 0.009, p = 0.92 in Toyama University]. However, significant differences were observed in education and estimated intelligence quotient (*t* (99) = 7.21, *p* = 4.71 x 10^−4^ and *t* (99) = 5.36, *p* = 6.10 x 10^−5^ in Nagoya University; *t* (94) = 9.86, *p* = 1.42 x 10^−15^ and *t* (90) = 5.84, *p* = 1.34 x 10^−7^ in Toyama University]. Significant differences were observed in age (*t* (98) = 8.81, p = 7.65 x 10^−14^), duration of illness (*t* (98) = 6.87, p = 1.29 x 10^−9^), and PANSS positive (*t* (96) = 2.1, *p* = 0.036) in the clinical data of patients between the two universities. All patients from Nagoya University and 42 of the 49 patients from Toyama University had been receiving antipsychotic medication (mean CPZ equivalent = 584.4 mg and 437.1 mg, respectively). There were no significant differences in the CPZ equivalent between universities (*t* (98) = 1.80, *p* = 0.076).

### SVM classification performance

Using the Nagoya University dataset as the training dataset, the ten-fold cross-validation classification accuracy of the training dataset was 73.3% (p-value = 2 x 10^−4^) (for the results of other measures, see Table 2). With the Toyama University dataset as the test dataset, the classification accuracy was 72.2% (p-value = 2x10^-4^) and AUC = 0.7402 (p-value = 0.0166) (for the results of other measures, see Table 3).

**Table 2:** Ten-fold cross validation classification performance of the trained support vector machine.

	Nagoya University model	Toyama University model
Training data set	Nagoya University	Toyama University
Accuracy	73.3%	72.2%
Sensitivity	64.0%	55.1%
Specificity	82.3%	89.6%
Positive predictive value	78.0%	84.4%
Negative predictive value	70.0%	66.2%

**Table 3:** Classification performance of the support vector machine using independent data set.

	Nagoya University model	Toyama University model
Test data set	Toyama University	Nagoya University
Accuracy	72.2%	72.3%
Sensitivity	61.2%	62.0%
Specificity	83.3%	82.4%
Positive predictive value	78.9%	77.5%
Negative predictive value	67.8%	68.9%

Using the Toyama University dataset as the training dataset, the ten-fold cross-validation classification accuracy of the training dataset was 72.2% (2 x 10^−5^) (for the results of other measures, see Table 2). The Nagoya University dataset was used to test the model. The classification accuracy was 72.3% (p-value = 0.0108) and AUC = 0.8043 (p-value = 0.0108) (for the results of other measures, see Table 3).

**Fig 1:**
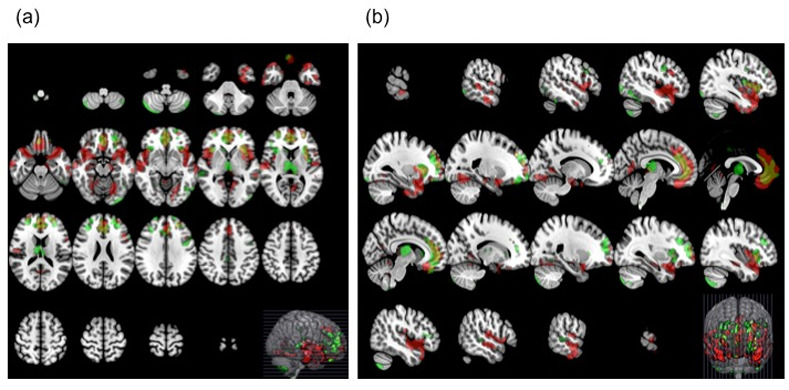
Regions with significant (FDR q <0.05 and cluster size of more than 100 voxels) weight values for both SVM models and their overlap. Regions in red represent area for the Nagoya University model, in green represent the Toyama University mode and yellow represent the overlap. Axial image (a) and sagittal image (b) (radiological convention).

Regions contributing significantly to the classification accuracy in the Nagoya University model included the bilateral insula, bilateral medial frontal cortex/anterior cingulate gyrus, bilateral frontal pole, bilateral parahippocampal gyrus/hippocampus, bilateral thalamus, bilateral lingual gyrus, fusiform gyrus, and left posterior cingulate gyrus. In contrast, regions with significant weights in the Toyama University model included the bilateral medial frontal cortex/anterior cingulate gyrus, bilateral insula, bilateral thalamus, bilateral frontal pole, cerebellum, bilateral middle frontal gyrus, right lingual gyrus, right middle temporal gyrus, and right caudate, among others. Regions where the significant weights of the two models overlapped were observed in the bilateral medial frontal cortex/anterior cingulate gyrus, bilateral frontal pole, bilateral frontal orbital cortex, bilateral middle/superior frontal gyrus, right superior temporal gyrus, bilateral insular cortex, and bilateral thalamus (Figs 1 and S1). The full list of regions with significant weights in the Nagoya University and Toyama University SVM models are given in Tables 4 and 5, respectively.

**Table 4:** Regions contributing to the classification performance of the trained support vector machine (Nagoya University model).

Cluster	Voxels	Center of Gravity			SVM Weights	Brain regions	Correlation with clinical data
Index		X (mm)	Y (mm)	Z (mm)	MEAN		
1	123	-6.63	-21.7	42.5	8.07E-05	Cingulate Gyrus, posterior division	
2	141	39.5	-21.8	-34.1	0.000103	R Temporal Fusiform Cortex	
3	143	7.6	-27.5	6.96	8.23E-05	R Thalamus	
4	151	5.39	-46.2	-37.6	0.0001	Cerebellar tonsils	PANSS ρ = -0.292, p = 0.040
5	269	-6.25	-30.7	1.73	8.53E-05	L Thalamus	duration year ρ = -0.320, p = 0.0223; CPZ ρ = -0.350, p = 0.013
6	286	31.9	36.8	-15.2	8.86E-05	R Frontal Pole /Frontal Orbital Cortex	
7	369	15.6	-52.8	-0.939	8.92E-05	R Lingual Gyrus	
8	396	-28	36.7	-16.7	8.87E-05	L Frontal Orbital Cortex /Frontal Pole	
9	425	32.1	-17.2	-15.8	9.06E-05	R Parahippocampal Gyrus / Hippocampus	
10	654	-15.9	-16.9	-19.6	9.48E-05	L Parahippocampal Gyrus / Hippocampus	PANSS ρ = -0.316, p = 0.025; PANSS positive ρ = -0.318, p = 0.025, PANSS negative ρ = -0.289, p = 0.042; CPZ ρ = -0.340, p = 0.016
11	705	19.3	-56.1	-14.3	9.57E-05	R Lingual Gyrus /Temporal Occipital Fusiform Cortex / Cerebellum	
12	731	28.3	60.3	4.04	8.53E-05	R Frontal Pole	
13	816	-7.21	-61.4	5.71	9.75E-05	L Lingual Gyrus	
14	1317	-27.1	52.7	15.1	8.77E-05	L Frontal Pole	
15	1452	-20.6	-69.4	-13.4	0.000111	L Occipital Fusiform Gyrus/Lingual Gyrus/Cerebellum	
16	7930	46.4	2	-11.1	0.000121	R Planum Polare /Insular Cortex /Temporal Pole	
17	9583	0.299	41.4	2.3	0.000114	Medial Frontal Gyrus/Cingulate Gyrus, anterior division	
18	10065	-41	4.73	-14.3	0.000126	L Temporal Pole /Insular Cortex /Planum Polare	

Abbreviations: Japanese version of the National Adult Reading Test (JART), chlorpromazine (CPZ), Positive and Negative Syndrome Scale (PANSS), Right (R), Left (L)

**Table 5:** Regions contributing to the classification performance of the trained support vector machine (Toyama University model).

Cluster	Voxels	Center of Gravity			SVM Weights	Brain regions	Correlation with clinical data
Index		X (mm)	Y (mm)	Z (mm)	MEAN		
1	117	24.6	37.9	-15.7	9.96E-05	R Frontal Pole/Frontal Orbital Cortex	
2	123	-60.5	-43.7	-2.86	9.61E-05	R Middle Temporal Gyrus, posterior division	
3	148	22.3	-57.3	-6.13	0.000107	R Lingual Gyrus/ Temporal Occipital Fusiform Cortex/Occipital Fusiform Gyrus	PANSS ρ = -0.325, p = 0.026
4	158	-32.1	-79.7	-50.4	0.000135	L Cerebelllum	
5	165	2.42	-31.5	40	9.52E-05	R Cingulate Gyrus, posterior division	
6	167	3.75	-47.5	-61	0.00012	R Cerebellum	onset age ρ = 0.314, p = 0.028
7	196	9.93	20.2	3.7	8.37E-05	R Caudate	
8	287	61.1	-32.1	2.3	9.55E-05	R Superior Temporal Gyrus, posterior division	
9	394	-42.2	-64.4	-51.4	0.000166	L Cerebelllum	
10	409	-48	6.45	32.1	0.000116	L Middle Frontal Gyrus	
11	525	34.1	35.7	26.9	0.00015	R Middle Frontal Gyrus	
12	773	-43.3	-75.5	-12.2	9.12E-05	L Lateral Occipital Cortex	
13	960	36.1	-74.5	-49.2	0.000144	R Cerebellum	
14	1079	35.6	22.2	1.6	0.00011	R Insular Cortex/Frontal Operculum Cortex	
15	1169	27.8	53.7	13.6	0.000116	R Frontal Pole	JART ρ = -0.311, p = 0.040
16	2259	1.57	-16.6	8.19	0.000114	R Thalamus	
17	10190	-10.1	40.8	4.95	0.00011	L Medial Frontal Cortex/Anterior Cingulate Gyrus	CPZ ρ = -0.339, p = 0.017

Abbreviations: Japanese version of the National Adult Reading Test (JART), chlorpromazine (CPZ), Positive and Negative Syndrome Scale (PANSS), Right (R), Left (L)

### Correlation between gray matter density in SVM-identified ROIs and clinical features

**Fig 2:**
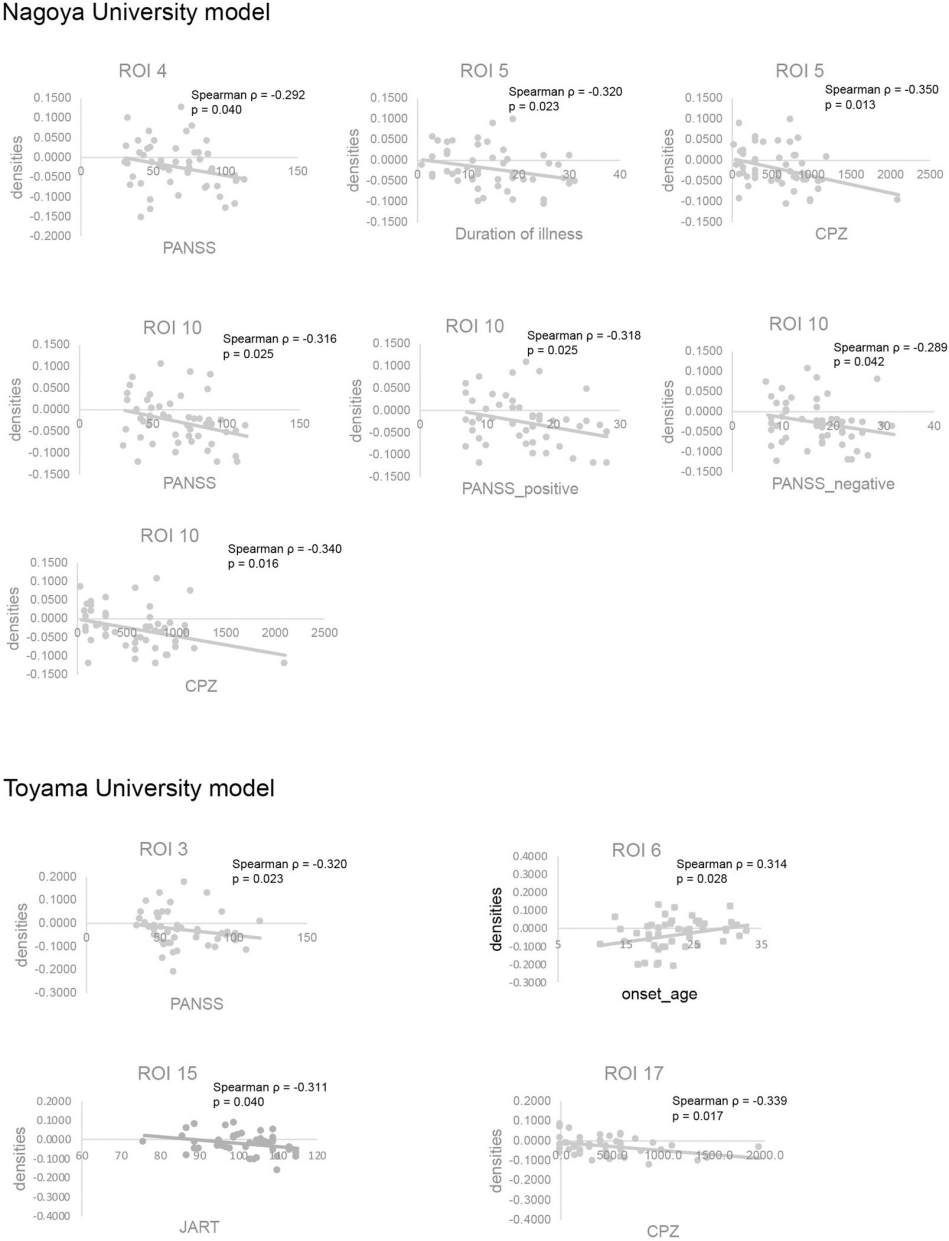
Correlation between gray matter densities in regions contributing significantly to the classification performance of the trained support vector machines and clinical features. Nagoya University model and Toyama University model (see also Tables 4 and 5). ROI, region-of-interest; PANSS, Positive and Negative Syndrome Scale; JART, Japanese version of the National Adult Reading Test; CPZ, chlorpromazine.

Tables 4 and 5 present the Montreal Neurological Institute coordinates of the center of gravity of all identified ROIs and the mean SVM weight values assigned to each in the Nagoya University and Toyama University SVM models, respectively. The significant correlations (p < 0.05, uncorrected) of mean gray matter density and clinical data are also presented in Tables 4 and 5 and Fig 2. All correlations are given in S1 and S2 Tables. There were no significant correlations after correcting for multiple comparisons using false discovery rate. Correlations of GM densities between ROIs are also shown in S3 and S4 Tables.

## Discussion

We used an SVM to identify the pattern of brain structural alterations that differentiates patients with schizophrenia from healthy controls. We demonstrated that both the Nagoya University and Toyama University SVM models had attained approximately the same classification accuracy in completely independent test datasets that used different MRI scanners, MRI protocols, and characteristics of patients. Our findings also revealed that the brain regions contributing to the classification included the areas frequently reported to be involved in the pathogenesis of schizophrenia. Most of the regions showed consistency across the two SVM models suggesting that these regions could potentially serve as robust neuroanatomical signatures of schizophrenia.

The classification performance accuracy of SVM models was >70% in both Nagoya and Toyama Universities despite the differences among MRI scanners and/or protocols. Although it is difficult to directly compare our results to those of previous studies due to differences in methodology and characteristics of the patients group, the classification accuracies of SVMs based on structural MRI findings have been reported to range from 63.2% to 91.8% [13]. Higher classification accuracy may be attained by optimizing the analysis method, standardizing the data protocol, using a single MRI scanner, and harmonizing participant selection. However, this might lead to reduced versatility and generalizability of the resulting SVM model. Feature selection has an effect on the classification accuracy and SVM training using preselected ROI analysis tends to have higher accuracy than voxel-based feature selection [13]. Although this approach could improve the accuracy, it could also exclude the potentially important brain regions differentiating the two groups [30]. In this study, we employed whole-brain analysis, which offered several advantages such as removal of bias in the ROI selection and identification of additional regions exhibiting morphological differences between patients with schizophrenia and healthy controls. Another factor to improve the accuracy is to standardize MRI data acquisition by using the same protocol and MRI scanner at a single site [31]. In clinical practice, inter-site variability in MRI scanners, MRI protocols, and clinical characteristics will remain. Therefore, SVM analysis using different datasets independently collected at different sites may have higher versatility and generalizability, which makes our study meaningful.

The performance of the SVMs was also assessed by sensitivity and specificity. The sensitivity was lower in our study than in previous studies. Previous studies on MVPA meta-analysis using structural MRI indicated a sensitivity of 76.4% (95% CI: 71.9–80.4%) and a specificity of 79.0% (95% CI: 74.6–82.8%) [7]. Our initial intention was to clarify the clinical characteristics of the misclassified patients; however, we did not succeed to specify the relation between patient clinical characteristics and misclassifications. Florkowski indicates that high sensitivity corresponds to high negative predictive value and is the ideal property of a “rule-out” test, while high specificity corresponds to high positive predictive value and is the ideal property of a “rule-in” test [32]. The prevalence of schizophrenia is approximately 1% of the population [1], therefore, high specificity (rule-in) rather than sensitivity (rule-out) might be more important for clinical application.

We detected highly similar patterns of regions contributing significantly to the classification accuracy of the trained SVMs in both models. Brain regions such as the bilateral medial frontal cortex, superior temporal cortex, and insula had structural brain alterations which were consistent with the previously reported structural brain alterations in schizophrenia patients by VBM analysis [3–5]. These patterns are also consistent with those identified in previous classification studies [11, 16, 17, 33]. Another region that was common in both models is the thalamus. Thalamic volume reductions have frequently been reported in studies employing VBM and ROI analyses; it was also reported in both those at high risk of schizophrenia and in relatives of patients with schizophrenia [5]. However, reports of thalamic volume reductions are relatively uncommon in studies applying SVM analyses [18, 34, 35].

On the other hand, there were also regions that were unique to each model, which may reflect the characteristics of the given site’s patient population. For instance, although regions in the lingual gyrus were present in both models, the identified regions did not overlap. The region associated with the Toyama University model (ROI 3) had GM density that also showed correlation to PANSS score (Fig 2), unlike the region associated with Nagoya University model. Alterations in the lingual gyrus/occipital fusiform in patients with schizophrenia have also been reported in previous SVM studies [11, 36, 37]. There are also a few VBM studies [4] and one ROI study [38] reporting gray matter density decreases in the occipital cortex in schizophrenic patients. The regions involved in the Nagoya University model are also more widespread in the cerebral cortex than that in the Toyama University model, which could be a reflection of illness duration or age considering that the patient group from Nagoya University were—on average—older and have longer duration of illness [39, 40]. In contrast, in the Toyama University model, the cerebellum has more widespread involvement than in the Nagoya University model. Moberget et al. [41] found in a large-scale international multisite study that cerebellar volume reduction in patients with schizophrenia was present already in the youngest patients. Cerebellar volume reduction in participants at high-risk of schizophrenia has been also reported using SVM analyses [10, 34, 35, 42–44].

Some of the brain regions contributing to the classification accuracy in this study are related to cognitive impairment and psychotic symptoms in patients with schizophrenia. The medial frontal cortex is related to executive function, and altered activity in the medial frontal cortex was reported during executive task performance [45]. The superior temporal gyrus plays an important role in auditory processing and language comprehension and is associated with positive symptoms in patients with schizophrenia [46]. The insular cortices are key nodes of the salience network, which has a central role in the detection of external and internal stimuli and the coordination of neural activity [47]. The salience network is related to reality distortion and psychotic symptoms such as delusions and hallucinations in patients with schizophrenia [48–50]. The lingual gyrus/occipital cortex belongs to the visual system. The impairment of visual processing in patients with schizophrenia has been frequently reported [51]. The cerebellum is a component of the cortico-cerebellar-thalamic-cortical circuit and may play a crucial role in the modulation of cortical activity. The cortico-cerebellar-thalamic-cortical circuit dysfunction is implicated in cognitive impairment and psychiatric symptoms in patients with schizophrenia [52]. Thus, the brain regions contributing to the classification accuracy may be associated with the pathophysiology of schizophrenia. The thalamus is a crucial node of the cortical-subcortical network and modulates information processing. Dysfunction of the thalamus is associated with cognitive impairment and abnormalities in a sensory experience such as hallucinations in patients with schizophrenia [53–55].

We investigated the correlation between the mean gray matter densities in the brain regions contributing to classification accuracy and clinical features. Our findings revealed that the gray matter densities of some regions were significantly correlated with the clinical characteristics of patients. Although the meaning of this observed association between some of the SVM-identified ROIs and clinical characteristics is unclear, this association demonstrates the efficacy of the SVM not only in classifying the two groups but also in identifying relevant discriminative features available in the data. In addition, some of the ROIs had gray matter densities that correlated with the amount of antipsychotic medication. These ROIs were observed in the thalamus and parahippocampal gyrus/hippocampus in Nagoya University model, and cingulate gyrus in Toyama University model. The effect of antipsychotics on gray matter changes is a matter of great concern and has been repeatedly reported [56, 57], but the reported effect on these regions is not consistent [58].

Finally, there are some limitations that should be mentioned in the interpretation of our results. Initially, in terms of methodology, we did not regress out the potential contribution of the differences in the total intracranial volume (ICV). Methodologically, we were concerned that the regression of the ICV would affect the performance of SVM. With regards to ICV in SVM analyses, in the literature, it is unclear whether ICV regression should be included [19, 31] or not [17, 59]. We did investigate whether the regression of ICV could affect the performance of SVM in our dataset. Our results showed a slight reduction in the classification accuracy (S6 Table in S1 File). More details are provided in S1 File. Second, some patients were using antipsychotics on the day of MRI scanning. Antipsychotic treatment is associated with gray matter density changes [60, 61]. Indeed, we demonstrated the effects of antipsychotics on reducing gray matter densities in the Toyama University model. Third, the sample size is relatively small. Nieuwenhuis et al. [17] reported that a large amount (n >130) of structural MRI data is required to distinguish patients with schizophrenia from controls. As mentioned above, although our SVM methods may not be applicable for diagnosis in clinical situations, they could be helpful for diagnosis of patients with schizophrenia and aid our understanding of the pathophysiology of brain alterations in patients with schizophrenia. In future studies, we need to use more sophisticated MRI data, for example, MRI data from drug-naïve first-episode patients [31], in order to secure more robust results.

In conclusion, our results indicated that the SVM performance remained consistent and accurate even when classifying data from different sites, with different MRI scanners, and different acquisition protocols. In addition, regions that consistently contribute to the classification were identified in both the SVM models, thus potentially providing a robust neuroanatomical signature of schizophrenia. Taken together, SVM could help in advancing our understanding of the pathophysiology of the structural changes accompanying schizophrenia, which could be one of the diagnostic findings of schizophrenia.

## Supporting information

S1 Fig Overlapping regions with significant (FDR q <0.05, and cluster size of more than 100 voxels) weight values between the Nagoya University and Toyama University SVM model. Axial image (a) and sagittal image (b); (radiological convention).
(TIF)

S1 Table Correlation of mean gray matter density in region-of-interest with clinical data (Toyama university model). (DOCX)

S2 Table Correlation of mean gray matter density in region-of-interest with clinical data (Nagoya university model). (DOCX)

S3 Table Correlation of mean gray matter density in region-of-interest (ROI) with other ROIs (Nagoya University model). (DOCX)

S4 Table Correlation of mean gray matter density in region-of-interest (ROI) with other ROIs (Toyama University model). (DOCX)

S1 File Supplemental analysis. (DOCX)
