# Supplementary figures and images for: Support vector machine-based classification of schizophrenia patients and healthy controls using structural magnetic resonance imaging from two independent sites
Source: PLoS One. 2020 Nov 24;15(11):e0239615. doi: 10.1371/journal.pone.0239615 (PMC7685428; doi:10.1371/journal.pone.0239615)

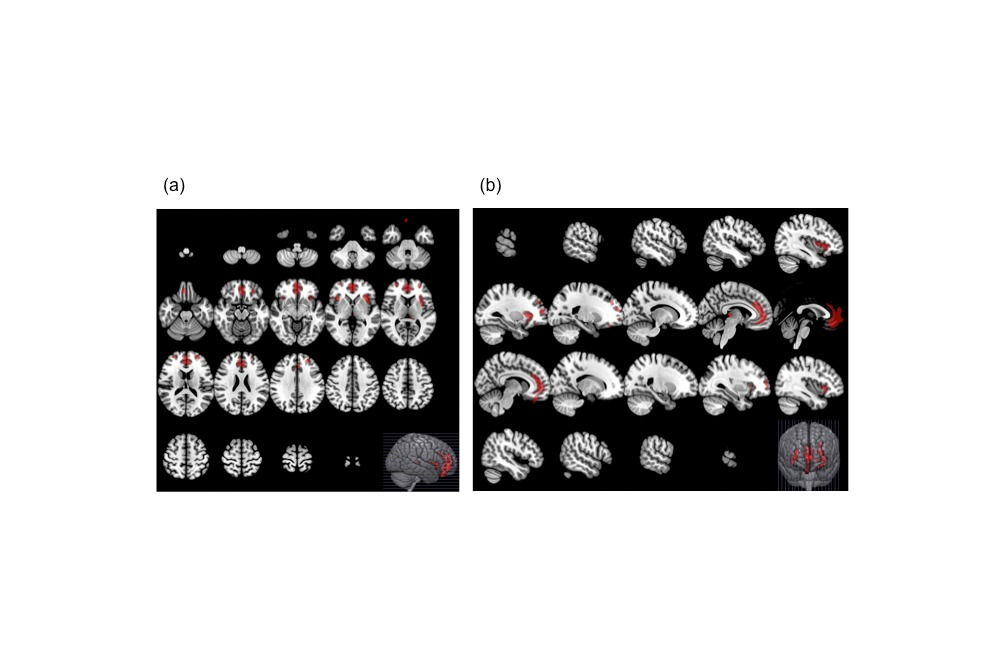

Supplement: S1 Fig — Axial image (a) and sagittal image (b); (radiological convention). (TIF) [file pone.0239615.s001.tif]
