# Supplementary material for: Support vector machine-based classification of schizophrenia patients and healthy controls using structural magnetic resonance imaging from two independent sites
Source: PLoS One. 2020 Nov 24;15(11):e0239615. doi: 10.1371/journal.pone.0239615 (PMC7685428; doi:10.1371/journal.pone.0239615)
Supplement: S1 Table — (DOCX) [file pone.0239615.s002.docx]

| **S1 Table. Correlation of mean gray matter density in region-of-interest with clinical data (Nagoya university model)** | | | | | | | |
| --- | --- | --- | --- | --- | --- | --- | --- |
|  | onset age | duration of illness | JART | PANSS | PANSS  positive | PANSS  negative | CPZ |
| ROI 1 | Spearman ρ = 0.003,  p = 0.981 | Spearman ρ = -0.125, p = 0.387 | Spearman ρ = 0.165,  p = 0.251 | Spearman ρ = -0.004, p = 0.981 | Spearman ρ = -0.025, p = 0.863 | Spearman ρ = 0.003,  p = 0.986 | Spearman ρ = 0.024,  p = 0.870 |
| ROI 2 | Spearman ρ = 0.073,  p = 0.614 | Spearman ρ = 0.063,  p = 0.661 | Spearman ρ = 0.048,  p = 0.740 | Spearman ρ = 0.028,  p = 0.849 | Spearman ρ = -0.025, p = 0.865 | Spearman ρ = 0.105,  p = 0.468 | Spearman ρ = 0.033,  p = 0.818 |
| ROI 3 | Spearman ρ = 0.247,  p = 0.083 | Spearman ρ = -0.265, p = 0.062 | Spearman ρ = 0.051,  p = 0.724 | Spearman ρ = -0.055,  p = 0.705 | Spearman ρ = -0.120, p = 0.406 | Spearman ρ = -0.045,  p = 0.759 | Spearman ρ = -0.155, p = 0.283 |
| ROI 4 | Spearman ρ = -0.020,  p = 0.889 | Spearman ρ = -0.054,  p = 0.708 | Spearman ρ = 0.104,  p = 0.472 | Spearman ρ = -0.292, p = 0.040 | Spearman ρ = -0.199, p = 0.165 | Spearman ρ = -0.266, p = 0.062 | Spearman ρ = -0.072, p = 0.619 |
| ROI 5 | Spearman ρ = 0.271,  p = 0.057 | Spearman ρ = -0.320,  p = 0.023 | Spearman ρ = 0.084,  p = 0.560 | Spearman ρ = -0.047,  p = 0.744 | Spearman ρ = -0.115,  p = 0.428 | Spearman ρ = -0.030, p = 0.837 | Spearman ρ = -0.350, p = 0.013 |
| ROI 6 | Spearman ρ = 0.033,  p = 0.819 | Spearman ρ = 0.022,  p = 0.881 | Spearman ρ = -0.094,  p = 0.516 | Spearman ρ = 0.105,  p = 0.468 | Spearman ρ = 0.075,  p = 0.604 | Spearman ρ = 0.016,  p = 0.911 | Spearman ρ = -0.042,  p = 0.770 |
| ROI 7 | Spearman ρ = 0.065,  p = 0.655 | Spearman ρ = -0.159,  p = 0.270 | Spearman ρ = 0.076,  p = 0.601 | Spearman ρ = -0.138, p = 0.338 | Spearman ρ = -0.090,  p = 0.532 | Spearman ρ = -0.122, p = 0.398 | Spearman ρ = 0.033,  p = 0.820 |
| ROI 8 | Spearman ρ = 0.043,  p = 0.769 | Spearman ρ = -0.018,  p = 0.900 | Spearman ρ = -0.032,  p = 0.825 | Spearman ρ = -0.052, p = 0.721 | Spearman ρ = -0.020, p = 0.888 | Spearman ρ = -0.136, p = 0.346 | Spearman ρ = -0.026, p = 0.858 |
| ROI 9 | Spearman ρ = 0.231,  p = 0.106 | Spearman ρ = -0.207,  p = 0.149 | Spearman ρ = 0.042,  p = 0.771 | Spearman ρ = -0.203, p = 0.158 | Spearman ρ = -0.176, p = 0.221 | Spearman ρ = -0.238, p = 0.095 | Spearman ρ = -0.197, p = 0.171 |
| ROI 10 | Spearman ρ = 0.054,  p = 0.712 | Spearman ρ = -0.066, p = 0.650 | Spearman ρ = 0.217,  p = 0.131 | Spearman ρ = -0.316, p = 0.025 | Spearman ρ = -0.318, p = 0.025 | Spearman ρ = -0.289, p = 0.042 | Spearman ρ = -0.340, p = 0.016 |
| ROI 11 | Spearman ρ = 0.089,  p = 0.539 | Spearman ρ = -0.137, p = 0.344 | Spearman ρ = 0.083,  p = 0.568 | Spearman ρ = -0.013, p = 0.929 | Spearman ρ = -0.084, p = 0.563 | Spearman ρ = 0.010,  p = 0.943 | Spearman ρ = -0.079, p = 0.585 |
| ROI 12 | Spearman ρ = -0.178,  p = 0.217 | Spearman ρ = 0.156,  p = 0.281 | Spearman ρ = 0.009,  p = 0.948 | Spearman ρ = -0.077,  p = 0.597 | Spearman ρ = -0.024, p = 0.867 | Spearman ρ = -0.154,  p = 0.287 | Spearman ρ = -0.043, p = 0.768 |
| ROI 13 | Spearman ρ = 0.099,  p = 0.495 | Spearman ρ = -0.052,  p = 0.718 | Spearman ρ = 0.166,  p = 0.248 | Spearman ρ = -0.176, p = 0.223 | Spearman ρ = -0.210, p = 0.143 | Spearman ρ = -0.049, p = 0.735 | Spearman ρ = -0.141, p = 0.330 |
| ROI 14 | Spearman ρ = -0.108, p = 0.454 | Spearman ρ = 0.129,  p = 0.370 | Spearman ρ = 0.019,  p = 0.893 | Spearman ρ = 0.017,  p = 0.905 | Spearman ρ = 0.019,  p = 0.898 | Spearman ρ = -0.044, p = 0.762 | Spearman ρ = -0.099, p = 0.495 |
| ROI 15 | Spearman ρ = 0.222,  p = 0.121 | Spearman ρ = -0.166,  p = 0.248 | Spearman ρ = -0.013,  p = 0.927 | Spearman ρ = -0.155,  p = 0.283 | Spearman ρ = -0.179,  p = 0.215 | Spearman ρ = -0.042, p = 0.773 | Spearman ρ = -0.082, p = 0.573 |
| ROI 16 | Spearman ρ = 0.064  p = 0.657 | Spearman ρ = -0.080,  p = 0.581 | Spearman ρ = -0.016, p = 0.915 | Spearman ρ = -0.151, p = 0.296 | Spearman ρ = -0.130, p = 0.368 | Spearman ρ = -0.179, p = 0.213 | Spearman ρ = -0.050, p = 0.732 |
| ROI 17 | Spearman ρ = -0.088  p = 0.545 | Spearman ρ = 0.102,  p = 0.479 | Spearman ρ = -0.034, p = 0.814 | Spearman ρ = -0.017, p = 0.905 | Spearman ρ = -0.031, p = 0.829 | Spearman ρ = -0.059,  p = 0.682 | Spearman ρ = -0.140, p = 0.333 |
| ROI 18 | Spearman ρ = 0.173  p = 0.230 | Spearman ρ = -0.135,  p = 0.351 | Spearman ρ = 0.000,  p = 0.999 | Spearman ρ = -0,151, p = 0.295 | Spearman ρ = -0.115, p = 0.425 | Spearman ρ = -0.161,  p = 0.265 | Spearman ρ = -0.145, p = 0.315 |
| Abbreviations: Region-of-interest (ROI), Japanese version of the National Adult Reading Test (JART), chlorpromazine (CPZ), Positive and Negative Syndrome Scale (PANSS) | | | | | | | |
