# Supplementary material for: Support vector machine-based classification of schizophrenia patients and healthy controls using structural magnetic resonance imaging from two independent sites
Source: PLoS One. 2020 Nov 24;15(11):e0239615. doi: 10.1371/journal.pone.0239615 (PMC7685428; doi:10.1371/journal.pone.0239615)
Supplement: S2 Table — (DOCX) [file pone.0239615.s003.docx]

| **S2 Table. Correlation of mean gray matter density in region-of-interest with clinical data (Toyama university model)** | | | | | | | |
| --- | --- | --- | --- | --- | --- | --- | --- |
|  | onset age | duration of illness | JART | PANSS | PANSS  positive | PANSS  negative | CPZ |
| ROI 1 | Spearman ρ = -0.028,  p = 0.848 | Spearman ρ = 0.042,  p = 0.774 | Spearman ρ = -0.279,  p = 0.066 | Spearman ρ = 0.047,  p = 0.752 | Spearman ρ = -0.236, p = 0.110 | Spearman ρ = 0.167,  p = 0.260 | Spearman ρ = 0.031,  p = 0.833 |
| ROI 2 | Spearman ρ = 0.018,  p = 0.902 | Spearman ρ = -0.036,  p = 0.807 | Spearman ρ = -0.010,  p = 0.948 | Spearman ρ = -0.275  p = 0.062 | Spearman ρ = -0.182, p = 0.221 | Spearman ρ = -0.221,  p = 0.136 | Spearman ρ = -0.094, p = 0.522 |
| ROI 3 | Spearman ρ = -0.102,  p = 0.485 | Spearman ρ = 0.198,  p = 0.172 | Spearman ρ = 0.195,  p = 0.204 | Spearman ρ = -0.325,  p = 0.026 | Spearman ρ = -0.153, p = 0.304 | Spearman ρ = -0.247, p = 0.094 | Spearman ρ = -0.035, p = 0.811 |
| ROI 4 | Spearman ρ = 0.090,  p = 0.538 | Spearman ρ = -0.260,  p = 0.071 | Spearman ρ = -0.120,  p = 0.440 | Spearman ρ = 0.136,  p = 0.361 | Spearman ρ = 0.025,  p = 0.866 | Spearman ρ = 0.112,  p = 0.453 | Spearman ρ = 0.112,  p = 0.445 |
| ROI 5 | Spearman ρ = -0.018,  p = 0.901 | Spearman ρ = 0.159,  p = 0.274 | Spearman ρ = 0.007  p = 0.963 | Spearman ρ = -0.073, p = 0.627 | Spearman ρ = -0.043, p = 0.775 | Spearman ρ = 0.049,  p = 0.745 | Spearman ρ = 0.001,  p = 0.994 |
| ROI 6 | Spearman ρ = 0.314,  p = 0.028 | Spearman ρ = -0.256  p = 0.076 | Spearman ρ = -0.171, p = 0.268 | Spearman ρ = 0.046  p = 0.757 | Spearman ρ = 0.044,  p = 0.768 | Spearman ρ = -0.016,  p = 0.916 | Spearman ρ = -0.034, p = 0.816 |
| ROI 7 | Spearman ρ = -0.010,  p = 0.945 | Spearman ρ = 0.144,  p = 0.323 | Spearman ρ = -0.017,  p = 0.912 | Spearman ρ = -0.001, p = 0.994 | Spearman ρ = -0.071, p = 0.635 | Spearman ρ = 0.120,  p = 0.423 | Spearman ρ = -0.135  p = 0.356 |
| ROI 8 | Spearman ρ = -0.112,  p = 0.443 | Spearman ρ = 0.087,  p = 0.553 | Spearman ρ = -0.011, p = 0.942 | Spearman ρ = -0.173, p = 0.245 | Spearman ρ = -0.246, p = 0.095 | Spearman ρ = -0.125, p = 0.403 | Spearman ρ = -0.040, p = 0.783 |
| ROI 9 | Spearman ρ = 0.059  p = 0.687 | Spearman ρ = -0.246, p = 0.089 | Spearman ρ = -0.178,  p = 0.248 | Spearman ρ = 0.069,  p = 0.644 | Spearman ρ = -0.007, p = 0.962 | Spearman ρ = 0.023,  p = 0.880 | Spearman ρ = 0.074,  p = 0.615 |
| ROI 10 | Spearman ρ = -0.036  p = 0.809 | Spearman ρ = 0.147,  p = 0.313 | Spearman ρ = -0.108,  p = 0.487 | Spearman ρ = -0.201, p = 0.176 | Spearman ρ = -0.193, p = 0.194 | Spearman ρ = -0.148, p = 0.321 | Spearman ρ = -0.122, p = 0.405 |
| ROI 11 | Spearman ρ = 0.026  p = 0.858 | Spearman ρ = -0.092,  p = 0.531 | Spearman ρ = -0.152,  p = 0.324 | Spearman ρ = -0.141, p = 0.346 | Spearman ρ = -0.050, p = 0.736 | Spearman ρ = -0.117, p = 0.432 | Spearman ρ = -0.167, p = 0.251 |
| ROI 12 | Spearman ρ = 0.002  p = 0.988 | Spearman ρ = 0.108,  p = 0.461 | Spearman ρ = -0.168,  p = 0.275 | Spearman ρ = -0.256,  p = 0.082 | Spearman ρ = -0.286, p = 0.051 | Spearman ρ = -0.103, p = 0.490 | Spearman ρ = -0.135, p = 0.356 |
| ROI 13 | Spearman ρ = 0.101  p = 0.490 | Spearman ρ = -0.137,  p = 0.349 | Spearman ρ = -0.043,  p = 0.784 | Spearman ρ = -0.118, p = 0.428 | Spearman ρ = -0.124, p = 0.405 | Spearman ρ = -0.150,  p = 0.315 | Spearman ρ = 0.006,  p = 0.967 |
| ROI 14 | Spearman ρ = 0.014  p = 0.922 | Spearman ρ = 0.028,  p = 0.849 | Spearman ρ = -0.222,  p = 0.148 | Spearman ρ = -0.075,  p = 0.616 | Spearman ρ = -0.045,  p = 0.763 | Spearman ρ = -0.005,  p = 0.971 | Spearman ρ = -0.192, p = 0.185 |
| ROI 15 | Spearman ρ = -0.096  p = 0.510 | Spearman ρ = 0.162,  p = 0.265 | Spearman ρ = -0.311, p = 0.040 | Spearman ρ = -0.215, p = 0.146 | Spearman ρ = -0.068, p = 0.648 | Spearman ρ = -0.185, p = 0.214 | Spearman ρ = -0.127, p = 0.383 |
| ROI 16 | Spearman ρ = -0.030  p = 0.835 | Spearman ρ = 0.111  p = 0.446 | Spearman ρ = -0.083, p = 0.592 | Spearman ρ = -0.257, p = 0.081 | Spearman ρ = -0.241, p = 0.103 | Spearman ρ = -0.171, p = 0.252 | Spearman ρ = -0.082, p = 0.574 |
| ROI 17 | Spearman ρ = -0.019  p = 0.899 | Spearman ρ = 0.103,  p = 0.483 | Spearman ρ = -0.130, p = 0.400 | Spearman ρ = -0.051, p = 0.732 | Spearman ρ = -0.080, p = 0.591 | Spearman ρ = 0.012,  p = 0.935 | Spearman ρ = -0.339, p = 0.017 |
| Abbreviations: Region-of-interest (ROI), Japanese version of the National Adult Reading Test (JART), chlorpromazine (CPZ), Positive and Negative Syndrome Scale (PANSS) | | | | | | | |
