# Supplementary material for: Support vector machine-based classification of schizophrenia patients and healthy controls using structural magnetic resonance imaging from two independent sites
Source: PLoS One. 2020 Nov 24;15(11):e0239615. doi: 10.1371/journal.pone.0239615 (PMC7685428; doi:10.1371/journal.pone.0239615)
Supplement: S3 Table — (DOCX) [file pone.0239615.s004.docx]

| **S3 Table. Correlation of mean gray matter density in region-of-interest (ROI) with other ROIs (Nagoya University Model)** | ROI 18 |  |  |  |  |  |  |  |  |  |  |  |  |  |  |  |  |  | 1.000 | Values indicated Spearman's correlation ρ; ** correlations is significant at the 0.01 level (2-tailed);* correlation is significant at the 0.05 level(2-tailed). | Abbreviations: Region-of-interest (ROI) |
| --- | --- | --- | --- | --- | --- | --- | --- | --- | --- | --- | --- | --- | --- | --- | --- | --- | --- | --- | --- | --- | --- |
|  | ROI 17 |  |  |  |  |  |  |  |  |  |  |  |  |  |  |  |  | 1.000 | 0.710** |  |  |
|  | ROI 16 |  |  |  |  |  |  |  |  |  |  |  |  |  |  |  | 1.000 | 0.793** | 0.843** |  |  |
|  | ROI 15 |  |  |  |  |  |  |  |  |  |  |  |  |  |  | 1.000 | 0.389** | 0.296* | 0.426** |  |  |
|  | ROI 14 |  |  |  |  |  |  |  |  |  |  |  |  |  | 1.000 | 0.041 | 0.600** | 0.715** | 0.519** |  |  |
|  | ROI 13 |  |  |  |  |  |  |  |  |  |  |  |  | 1.000 | 0.209 | 0.605** | 0.520** | 0.483** | 0.490** |  |  |
|  | ROI 12 |  |  |  |  |  |  |  |  |  |  |  | 1.000 | 0.288* | 0.849** | 0.121 | 0.663** | 0.771** | 0.536** |  |  |
|  | ROI 11 |  |  |  |  |  |  |  |  |  |  | 1.000 | 0.093 | 0.581** | 0.094 | 0.680** | 0.386** | 0.365** | 0.450** |  |  |
|  | ROI 10 |  |  |  |  |  |  |  |  |  | 1.000 | 0.381** | 0.281* | 0.528** | 0.178 | 0.389** | 0.546** | 0.565** | 0.643** |  |  |
|  | ROI 9 |  |  |  |  |  |  |  |  | 1.000 | 0.666** | 0.411** | 0.265 | 0.441** | 0.246 | 0.445** | 0.672** | 0.441** | 0.670** |  |  |
|  | ROI 8 |  |  |  |  |  |  |  | 1.000 | 0.538** | 0.417** | 0.336* | 0.616** | 0.306* | 0.542** | 0.311* | 0.665** | 0.639** | 0.636** |  |  |
|  | ROI 7 |  |  |  |  |  |  | 1.000 | 0.059 | 0.248 | 0.217 | 0.691** | 0.108 | 0.556** | 0.015 | 0.553** | 0.252 | 0.193 | 0.267 |  |  |
|  | ROI 6 |  |  |  |  |  | 1.000 | 0.160 | 0.702** | 0.540** | 0.326* | 0.412** | 0.642** | 0.296* | 0.606** | 0.369** | 0.695** | 0.636** | 0.638** |  |  |
|  | ROI 5 |  |  |  |  | 1.000 | 0.318* | 0.105 | 0.258 | 0.470** | 0.340* | 0.304* | 0.254 | 0.239 | 0.281* | 0.155 | 0.452** | 0.321* | 0.522** |  |  |
|  | ROI 4 |  |  |  | 1.000 | 0.423** | 0.158 | -0.040 | 0.139 | 0.164 | 0.022 | 0.072 | 0.211 | -0.093 | 0.210 | -0.044 | 0.229 | 0.090 | 0.188 |  |  |
|  | ROI 3 |  |  | 1.000 | 0.223 | 0.676** | 0.172 | -0.036 | 0.311* | 0.227 | 0.318* | 0.178 | 0.290* | 0.150 | 0.326* | 0.024 | 0.373** | 0.420** | 0.528** |  |  |
|  | ROI 2 |  | 1.000 | 0.208 | 0.156 | 0.175 | 0.030 | -0.013 | 0.203 | 0.099 | 0.232 | 0.052 | 0.187 | 0.019 | 0.166 | 0.181 | 0.177 | 0.251 | 0.270 |  |  |
|  | ROI 1 | 1.000 | 0.150 | 0.478** | 0.197 | 0.380** | 0.449** | 0.113 | 0.415** | 0.388** | 0.433** | 0.300* | 0.417** | 0.410** | 0.346* | 0.278 | 0.662** | 0.600** | 0.617** |  |  |
|  |  | ROI 1 | ROI 2 | ROI 3 | ROI 4 | ROI 5 | ROI 6 | ROI 7 | ROI 8 | ROI 9 | ROI 10 | ROI 11 | ROI 12 | ROI 13 | ROI 14 | ROI 15 | ROI 16 | ROI 17 | ROI 18 |  |  |
