# Supplementary material for: Support vector machine-based classification of schizophrenia patients and healthy controls using structural magnetic resonance imaging from two independent sites
Source: PLoS One. 2020 Nov 24;15(11):e0239615. doi: 10.1371/journal.pone.0239615 (PMC7685428; doi:10.1371/journal.pone.0239615)
Supplement: S4 Table — (DOCX) [file pone.0239615.s005.docx]

| **S4 Table. Correlation of mean gray matter density in region-of-interest (ROI) with other ROIs (Toyama University Model)** | ROI 17 |  |  |  |  |  |  |  |  |  |  |  |  |  |  |  |  | 1.000 | Values indicated Spearman's correlation ρ; ** correlations is significant at the 0.01 level (2-tailed);* correlation is significant at the 0.05 level (2-tailed) | Abbreviations: Region-of-interest (ROI) |
| --- | --- | --- | --- | --- | --- | --- | --- | --- | --- | --- | --- | --- | --- | --- | --- | --- | --- | --- | --- | --- |
|  | ROI 16 |  |  |  |  |  |  |  |  |  |  |  |  |  |  |  | 1.000 | 0.209 |  |  |
|  | ROI 15 |  |  |  |  |  |  |  |  |  |  |  |  |  |  | 1.000 | 0.209 | 0.649** |  |  |
|  | ROI 14 |  |  |  |  |  |  |  |  |  |  |  |  |  | 1.000 | 0.549** | 0.116 | 0.704** |  |  |
|  | ROI 13 |  |  |  |  |  |  |  |  |  |  |  |  | 1.000 | 0.232 | 0.067 | 0.254 | 0.192 |  |  |
|  | ROI 12 |  |  |  |  |  |  |  |  |  |  |  | 1.000 | 0.191 | 0.254 | 0.232 | 0.288* | 0.426** |  |  |
|  | ROI 11 |  |  |  |  |  |  |  |  |  |  | 1.000 | 0.164 | -0.052 | 0.289* | 0.540** | 0.113 | 0.262 |  |  |
|  | ROI 10 |  |  |  |  |  |  |  |  |  | 1.000 | 0.521** | 0.107 | 0.174 | 0.287* | 0.408** | 0.058 | 0.327* |  |  |
|  | ROI 9 |  |  |  |  |  |  |  |  | 1.000 | 0.116 | -0.052 | 0.205 | 0.890** | 0.265 | 0.101 | 0.241 | 0.173 |  |  |
|  | ROI 8 |  |  |  |  |  |  |  | 1.000 | 0.219 | 0.326* | 0.103 | 0.486** | 0.364* | 0.164 | 0.257 | 0.249 | 0.482** |  |  |
|  | ROI 7 |  |  |  |  |  |  | 1.000 | 0.209 | -0.102 | 0.129 | 0.196 | 0.139 | -0.080 | 0.088 | 0.148 | 0.087 | 0.173 |  |  |
|  | ROI 6 |  |  |  |  |  | 1.000 | 0.020 | 0.101 | 0.698** | -0.034 | -0.068 | 0.204 | 0.694** | 0.241 | 0.070 | 0.027 | 0.199 |  |  |
|  | ROI 5 |  |  |  |  | 1.000 | 0.136 | 0.112 | 0.292* | -0.105 | 0.057 | 0.195 | 0.466** | -0.066 | 0.207 | 0.281 | -0.077 | 0.433** |  |  |
|  | ROI 4 |  |  |  | 1.000 | 0.018 | 0.579** | -0.060 | 0.205 | 0.840** | -0.022 | 0.018 | 0.209 | 0.746** | 0.216 | 0.044 | 0.168 | 0.130 |  |  |
|  | ROI 3 |  |  | 1.000 | 0.011 | 0.421** | 0.070 | 0.049 | 0.419** | -0.012 | 0.047 | 0.019 | 0.300* | 0.121 | 0.138 | 0.158 | 0.186 | 0.322* |  |  |
|  | ROI 2 |  | 1.000 | 0.439** | 0.186 | 0.284* | 0.233 | 0.194 | 0.652** | 0.127 | 0.281 | 0.190 | 0.292* | 0.318* | 0.253 | 0.315* | 0.191 | 0.498** |  |  |
|  | ROI 1 | 1.000 | 0.266 | 0.181 | 0.131 | 0.133 | 0.172 | 0.240 | 0.458** | 0.186 | -0.016 | 0.044 | 0.340* | 0.182 | 0.315* | 0.448** | 0.211 | 0.402** |  |  |
|  |  | ROI 1 | ROI 2 | ROI 3 | ROI 4 | ROI 5 | ROI 6 | ROI 7 | ROI 8 | ROI 9 | ROI 10 | ROI 11 | ROI 12 | ROI 13 | ROI 14 | ROI 15 | ROI 16 | ROI 17 |  |  |
