# Supplementary material for: Support vector machine-based classification of schizophrenia patients and healthy controls using structural magnetic resonance imaging from two independent sites
Source: PLoS One. 2020 Nov 24;15(11):e0239615. doi: 10.1371/journal.pone.0239615 (PMC7685428; doi:10.1371/journal.pone.0239615)
Supplement: S1 File — (DOCX) [file pone.0239615.s006.docx]

Supplementary Analysis

We performed supplementary analyses with the total intracranial volume (ICV) regressed out in addition to age and sex to investigate how this preprocessing step would affect the classification performance of SVM. We estimated the ICV by the addition of gray matter (GM), white matter (WM), and cerebrospinal fluid (CSF) volumes, which were automatically calculated during preprocessing. Table S5 shows the means (SD) of ICV for the patients and healthy controls at each site. Patients and controls did not differ significantly within each university [*t* (99) = 1.916, *p* = 0.058 in Nagoya University; *t* (95) =1.281, *p* = 0.203 in Toyama University].

S5 Table. Mean total intracranial volume

|  | **Nagoya University** | | **Toyama University** | |
| --- | --- | --- | --- | --- |
|  | **SCZ (*n* = 50)** | **CON (*n* = 51)** | **SCZ (*n* = 49)** | **CON (*n* = 48)** |
| ICV (cm3) | 1406.7 (143.7) | 1459.4 (132.9) | 1369.4 (151.8) | 1404.1 (111.7) |

The results of the supplementary analyses are summarized in Table S6 showing the ten-fold cross-validation classification accuracy of the training data set and of the test data set. By regressing out ICV, a slight reduction in the performance of the SVM can be observed. We do note that as ICV was calculated as the total volume of GM, WM, and CSF, its value could be influenced by changes in GM volume specifically in the patient population where the GM alterations could be widespread. This, in turn, could potentially reduce the difference between controls and patients after ICV regression.

S6 Table. SVM classification performance after regressing out the total intracranial volume in addition to age and sex

|  | Nagoya University model | Toyama University model |
| --- | --- | --- |
| Training data set | 74.3% | 70.1% |
| Test data set | 66.0% | 69.3% |
